# Proapoptotic Activity of *Achillea membranacea* Essential Oil and Its Major Constituent 1,8-Cineole against A2780 Ovarian Cancer Cells

**DOI:** 10.3390/molecules25071582

**Published:** 2020-03-30

**Authors:** Ashraf N. Abdalla, Usama Shaheen, Qasem M. A. Abdallah, Guido Flamini, Majdi M. Bkhaitan, Mohamed I. S. Abdelhady, Roberta Ascrizzi, Ammar Bader

**Affiliations:** 1 Faculty of Pharmacy, Umm Al-Qura University, Makkah 21955, Saudi Arabia; anabdrabo@uqu.edu.sa (A.N.A.); usamayosef2003@yahoo.com (U.S.); bariumchloride@hotmail.com (M.M.B.); mohibrahem@yahoo.com (M.I.S.A.); 2 Department of Pharmacognosy, Faculty of Pharmacy, Al-Azhar University, Cairo 11651, Egypt; 3 Department of Pharmacology and Toxicology, College of Pharmacy, Taif University, Taif, Makkah 21974, Saudi Arabia; qasem79@yahoo.com; 4 Department of Pharmacology & Biomedical Sciences, Faculty of Pharmacy and Medical Sciences, University of Petra, Amman 11196, Jordan; 5 Department of Pharmacy, University of Pisa, Via Bonanno 6, 56126 Pisa, Italy; roberta.ascrizzi@gmail.com; 6 Basic Medical Sciences Unit, Arab American University, Jenin 240, Palestine; 7 Department of Pharmacognosy, Faculty of Pharmacy, Helwan University, Cairo 11651, Egypt

**Keywords:** *Asteraceae*, essential oil, GC-EI-MS, 1,8-cineole, cytotoxicity, apoptosis, cell cycle

## Abstract

Among the hundreds of reported *Achillea* species, *A. membranacea* (Labill.) DC. is one of the six that grow in Jordan. Many species of this genus are used in folk medicine to treat a variety of ailments and several biological and pharmacological activities have been ascribed to their essential oil (EO). For this study, the EO obtained from a specimen of *A. membranacea* grown in Jordan was analyzed by GC-MS. Ninety-six compounds were detected, of which oxygenated monoterpenes was the predominant class (47.9%), followed by non-terpene derivatives (27.9%), while sesquiterpenes represented 14.2% of the total composition. The most abundant compound in the EO was 1,8-cineole (21.7%). The cytotoxic activity of the EO was evaluated against three cancer cell lines (MCF7, A2780 and HT29), and one normal fibroblast cell line (MRC5) by MTT assay. Significant growth inhibition was observed in EO-exposed A2780 and HT29 cells (IC_50_ = 12.99 and 14.02 μg/mL, respectively), while MCF7 and MRC5 were less susceptible. The EO induced apoptosis and increased the preG1 events in A2780 cells. 1,8-Cineole, the major constituent of the EO, exhibited submicromolar cytotoxicity against A2780 cells, and was 42 times more selective against MRC5 cells. Its cytotoxicity against A2780 cells was comparable with that of doxorubicin, but 1,8-cineole was more selective for MRC5 normal cells. Interestingly, 1,8-cineole enhanced apoptosis in A2780, and caused a remarkable dose-dependent increase in preG1 events. Thus, 1,8-cineole has demonstrated promising cytotoxic and proapoptotic properties.

## 1. Introduction

Medicinal plants and their constituents have countless biological properties, based mainly on their ability to interact with key enzymes linked to several ailments, including: cancer, diabetes, inflammation, hypertension and Alzheimer’s disease [1,2,3,4,5]. Recently, EOs have caught the attention of scientists due to their ability to prevent and treat cancer, as well as their state-of-the-art application in the most advanced and sophisticated technologies in nanoscience [6,7,8].

The genus *Achillea* includes over a hundred species and subspecies, which are distributed in the Northern hemisphere [9]. The use of the inflorescence, as well as the whole plant, of many of these species has a long-established role in folk medicine to treat a wide range of ailments that mainly affect the digestive system. It takes various application forms, which differ depending on the geographical area [10,11,12,13]. Besides their medicinal properties, some *Achillea* species are also cultivated as ornamental plants.

*A. membranacea* (Labill.) DC. is one of the six species reported in Jordan [14]: it is distributed in the Irano-Turanian phytogeographic region, which includes Jordan, Palestine, Syria, Lebanon, Iraq and Turkey [15]. *A. membranacea* is a perennial herb. It has numerous stems (25–50 cm), is lignified in the basal section, exhibits pannose, linear, sessile and pinnatisect (1.5–6 cm × 0.3–0.6 cm) leaves, and has a capitula up to 20 mm and yellow ray florets [15].

The EO of many *Achillea* species possesses several biological and pharmacological activities. It is antimicrobial, antimalarial, insecticidal, phytotoxic, anti-inflammatory and antihemorrhoidal [16,17,18,19,20]. Anticancer and cytotoxic effects were reported for the extracts of some *Achillea* species, including *A. clavennae* L. and *A. millefolium* L., the iso-seco-guaianolide, which was isolated from *A. clavennae* L., exhibited potent antiproliferative and apoptotic effects on U251 and glioma cell lines, with a potency comparable to that of cisplatin [21], mediated by its oxidative and mitotic activities. Another compound, casticin, showed multiple antitumor activities. Casticin, which was isolated from *A. millefolium* L. through HPLC, showed cytotoxic, early apoptosis, G_2_/M cell cycle arrest and consequently disruption of the mitotic spindle in two MCF7 sublines [22]. In a previous study, the methanol extract of *A. membranacea* exerted a moderate cytotoxic effect upon Jurkat cells (T-cell leukemia) using the MTT assay [23].

As a part of an ongoing research program on the Middle Eastern plants bioactivity [24], we have selected *A. membranacea* for this study with the aim to analyze the composition of the EO of *A. membranacea* collected in Jordan and to evaluate its cytotoxic activity against breast (MCF7), ovary (A2780) and colon (HT29) cancer cell lines, and non-tumorigenic human fetal lung fibroblasts (MRC5 cells). Moreover, its effects on apoptosis and cell cycle effect of the EO on A2780 cells were investigated. Beside the EO, we have tested the cytotoxicity, induction of apoptosis and cell cycle effect of its major constituent, 1,8-cineole, against A2780 cell line, disclosing the possible action mechanism of the EO and its major constituent.

## 2. Results

### 2.1. A. membranacea Essential Oil Composition

The complete composition of the essential oil (EO) hydrodistilled from *A. membranacea* aerial parts is reported in Table 1. A total of 96 compounds were identified, accounting for 92.0% of the total composition, which was chiefly made up of monoterpenes. In particular, over 45% of the total was constituted by oxygenated monoterpenes, of which 1,8-cineole was the most represented: exhibiting a relative abundance of 21.7%, it was also the most abundant compound in the EO. Other quantitatively relevant compounds of this chemical class were borneol and *trans*-pinocarveol, whose relative concentrations were 4.3% and 3.1%, respectively. Non-terpene derivatives accounted for up to 22.4%, making them the second most abundant detected chemical class of compounds in the EO. Among them, aldehydes prevailed (12.7%), with decanal and (*Z*)-4-decenal being the most abundant; hydrocarbons followed, accounting for up to 5.0%. Sesquiterpenes were almost equally represented by their hydrocarbon and oxygenated forms (6.7% and 6.9%, respectively). Among the former, α-cadinene was the most represented, while spathulenol was the most abundant of the latter class. Apocarotenes and phenylpropanoids followed, with relative concentrations of 4.2% and 3.9%, respectively.

### 2.2. Cytotoxic Activity

The cytotoxic activity of *A. membranacea* EO against the tested cell lines is reported in Table 2. It induced significant growth inhibition in ovarian (A2780) and colorectal (HT29) cells (IC_50_ = 12.99 and 14.02 μg/mL, respectively). Negligible toxicity was noticed, however, in treated breast cancer (MCF7) and human fetal lung fibroblast (MRC5) cells (IC_50_ = 50.86 μg/mL and IC_50_ = 49.25 μg/mL, respectively). The EO was four times more selective for A2780 and HT29 cell lines compared to MRC5 cells (IC_50_ = 49.25 μg/mL). The major component, 1,8-cineole, was selected for further cytotoxicity assessments. 1,8-Cineole showed remarkable cytotoxicity compared to *A. membranacea* EO in A2780 cells (0.26 μM); its selectivity was 42 times higher for A2780 compared to MRC5 cells (10.5 μM). Using the same cell lines, the selectivity profile of 1,8-cineole was better compared to doxorubicin, as the latter showed only a twofold improvement in selectivity (Table 3).

### 2.3. Induction of Cell Apoptosis

**Figure 1:**
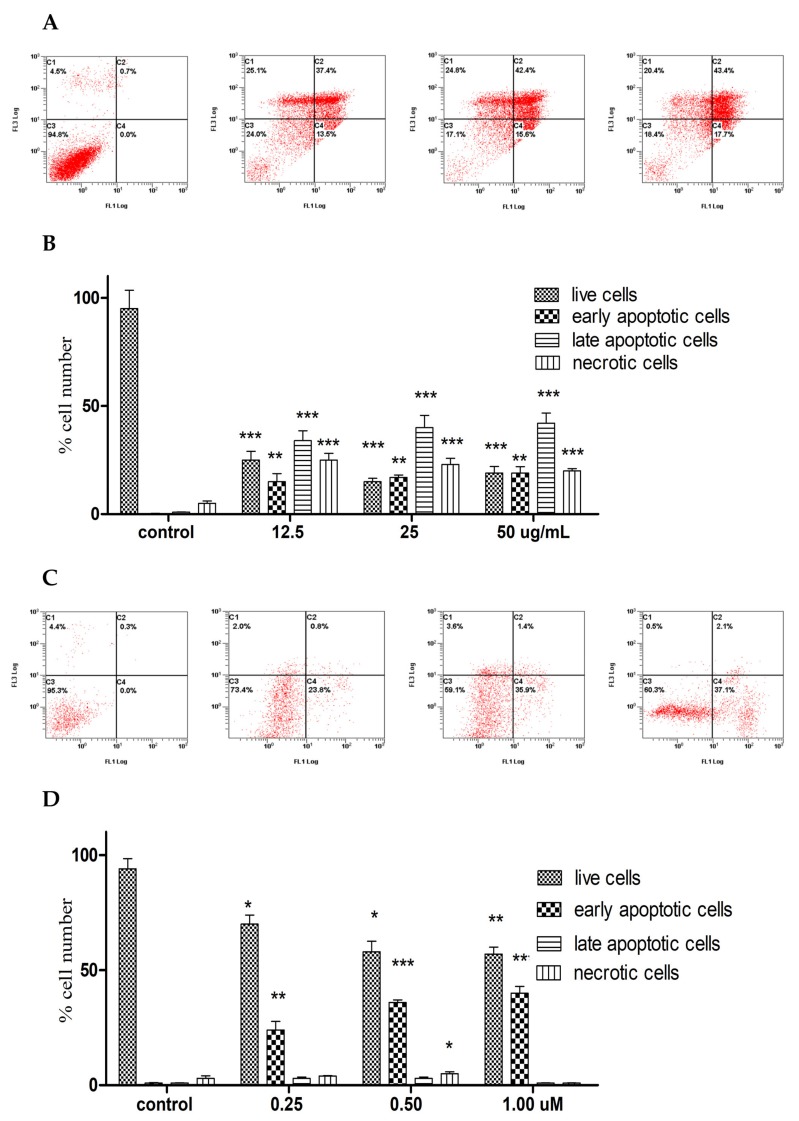
Detection of early and late apoptosis in A2780 cells (72 h) treated with *A. membranacea* essential oil (**A**,**B**), and 1,8-cineole (**C**,**D**). Dot histograms (**A**,**C**): x-axis: annexin V, y-axis: propidium iodide. C1: necrotic cells, C2: late apoptotic cells, C3: live cells, C4: early apoptotic cells. Data shown are % mean ± SD (*n* = 3). All experiments were performed three times. Statistical differences, compared to untreated control cells, were assessed by a one-way ANOVA with the Tukey’s post-hoc multiple comparison test. *p* < 0.05 (*), *p* < 0.01 (**) and *p* < 0.001 (***) were taken as significant.

In order to explore the mechanism of action, the induction of apoptosis by *A. membranacea* EO on A2780 cells was investigated at the same time-point of testing the cytotoxicity (72 h) to confirm its apoptotic *vs* necrotic properties. The EO showed a dose-dependent increase of combined early and late apoptosis in A2780 cells, although its effect at the 25 and 50 µg/mL was close. The EO caused mainly a late apoptosis compared to early apoptosis in A2780 cells. There was also an increase in the necrotic events from 4% to 20% at the three doses (Figure 1A,B). The situation was inverted when 1,8-cineole was used alone in A2780 cells at the same time-point, as it caused a dose-dependent increase in early apoptosis up to 37% at 1 µM, with only 2% late apoptosis and negligible necrotic effect (Figure 1C,D).

### 2.4. Perturbation of the Cell Cycle

**Figure 2:**
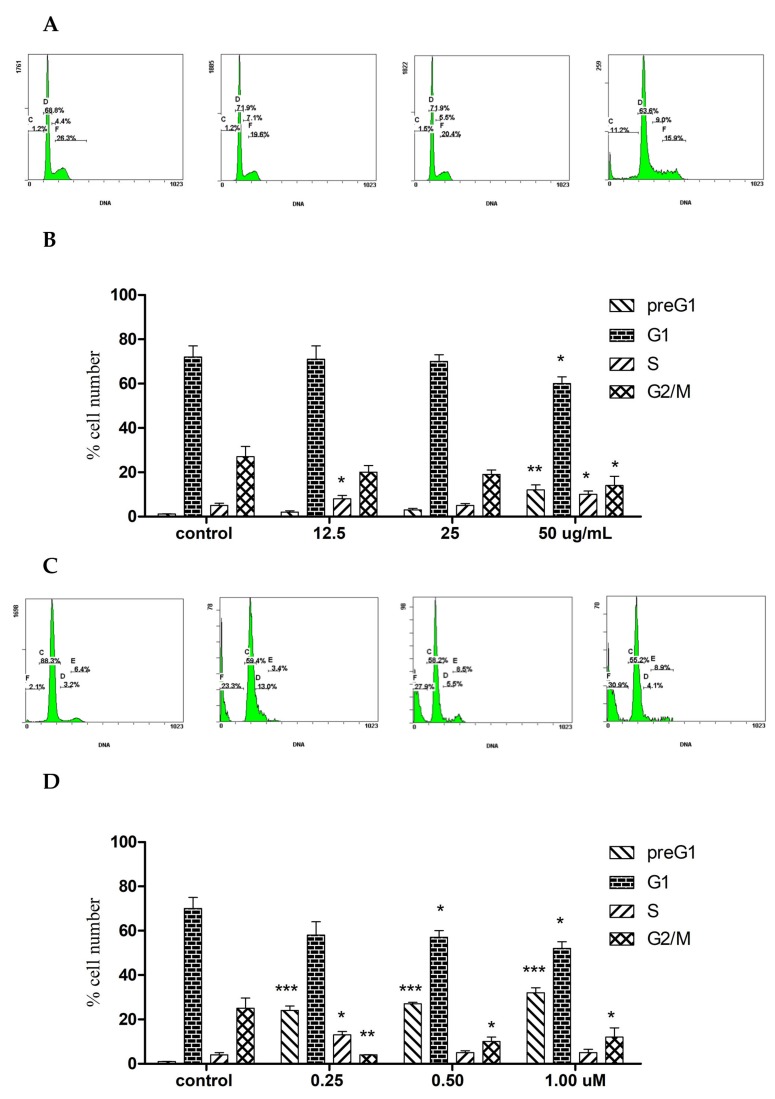
Flow cytometry showing the effect of *A. membranacea* EO (**A**,**B**) and 1,8-cineole (**C**,**D**) on cell cycle distribution after 72 h treatment in A2780 cells. A, C: X-axis: DNA content of 20,000 events, y axis: % cell number (*n* = 3, experiments were repeated 3×). Statistical differences, compared to untreated control cells, were assessed by a one-way ANOVA with the Tukey’s post-hoc multiple comparison test. *p* < 0.05 (*), *p* < 0.01 (**) and *p* < 0.001 (***) were taken as significant.

The cell-cycle analysis on *A. membranacea* EO treated A2780 cells was performed (72 h), showing a non-dose dependent slight S arrest at 12.5 and 50 μg/mL. Moreover, the EO caused a 10% increase in the preG1 at the expense of G_1_ and G_2_/M decreased events (Figure 2A,B). 1,8-Cineole also caused S phase arrest only at 12.5 μg/mL, but it induced a 15-20-fold sharp dose dependent increase in the preG_1_ apoptotic events in A2780 cells (Figure 2C,D).

## 3. Discussion

The main constituent of the *A. membranacea* EO analyzed in this study was 1,8-cineole (over 20% of the total). This monoterpene was also found to be a major constituent of some other *Achillea* spp., such as *A. santolina* L. (syn. of *Achillea tenuifolia* Lam.; 16.7%) [25], *A. tomentosa*L. (56.1%) [26], *A. wilhelmsii* (syn. of *Achillea santolinoides* subsp*. wilhelmsii* (K.Koch) Greuter), *A. thracica* Velen. (7.46%–35.72%) [27] and *A. millefolium* L. (1.2%–19.8%) [28]. Previous studies on 1,8 cineole, or on EOs containing it, reported apoptotic effects on a variety of cancer cell lines, including: SK-MEL-28 (human melanoma); A549 (human lung carcinoma); Colo-205 (Human Caucasian colon adenocarcinoma); (SiHa) cells (human cervical carcinoma); Hep-G2 (hepatocellular carcinoma); MCF-7, T47D, MDA-MB-231 (human breast adenocarcinoma); RKO (Human colon carcinoma); Caco-2 (human Caucasian colon adenocarcinoma); A431 (squamous cell carcinoma); MG-63 (osteosarcoma) and P815 (murine mastocytoma) [29,30,31,32,33,34,35,36,37]. This study has enriched the list of those cancer cell lines susceptible to 1,8-cineole and EO rich in1,8-cineole, and has demonstrated its potent effect on the ovarian cancer cell line (A2780).

In a previous published study, we reported the marginal cytotoxic effect of the methanol extract of the aerial parts of *A. membranacea* on Jukart cells, with an IC_50_ value of 60 ± 5.1 μg/mL [15]. That extract, however, did not exert any cytotoxic effect on MCF7 or A2780 cancer cell lines (IC_50_ > 200 μg/mL) [23]. The growth of A2780 cells in this study was, however, significantly inhibited (IC_50_ = 12.99 μg/mL) by *A. membranacea* EO, since the natural product components are generally considered cytotoxic when their IC_50_ is ≤ 20 μg/mL [38]. The tested EO resulted in the cytotoxicity of the A2780 cell line, while its effect on MCF7was negligible (IC_50_ = 50.86 μg/mL). In this study, both the EO and 1,8-cineole induced apoptosis in A2780 cells; but 1,8-cineole induced more early apoptosis with minimal late apoptosis and necrosis, compared to a major induction of late apoptosis by the EO at the same time point. The EO also caused 4%–20% necrotic events in A2780 cells compared to control cells, indicating the possible effect of other components. The improved cytotoxic and apoptotic activities of 1,8-cineole compared to *A. membranacea* indicates the possible involvement of one major mechanism of action in *A. membranacea* EO*,* while the substantial difference between the activities of the EO and the methanol extract of the aerial parts could be due to the difference in the constituents of each extract.

Furthermore, the cell cycle perturbation results of the present work were similar to those observed in a previous study conducted on wild-type p53 H460 cells (but not mutant p53 H460 cells) after the treatment with *Achillea millefolium* hydroalcoholic extract [39]. Pereira et al. (2018) demonstrated the induction of apoptosis and S-phase arrest, with an increased level of p53 and p21 indicating the different mechanisms involved in the *Achillea millefolium* extract-induced growth arrest. Likewise, a previous study reported that casticin, a flavonoid extracted from *Achillea millefolium*, caused G_2_/M arrest in HCT116 colon cancer cells, which was confirmed by the induction of p21 and down-regulation of CDK1[14]. However, 1,8 cineole was reported to induce cell cycle arrest in the G_2_/M phase in the HCT116 cell line [30], while the studied *A. membranacea* EO and 1,8 cineole induced preG_1_ increase, which indicate DNA fragmentation and confirm the pro-apoptotic effect in A2780 cells. Further mechanistic studies of the activities of *A. membranacea* components and 1,8 cineole against an array of cancer cell lines are worthwhile, as well as *in vivo* studies, which will provide more support for their application.

## 4. Materials and Methods

### 4.1. Plant Material

The aerial parts of *A. membranacea* were collected during the spring in the Dab’a desert reserve (50 km South of Amman), Jordan (GPS coordinates: 31° 31’ 58” N, 36° 01’ 20” E). The plant was identified by Prof. A. Bader. A voucher specimen was deposited in the herbarium of the Pharmacognosy Lab (Number Jo-It 2018/2), at Umm Al-Qura University, Makkah, Saudi Arabia.

### 4.2. Hydrodistillation of the Essential Oil

The hydrodistillation of *A. membranacea* was performed with a Clevenger-type apparatus equipped with an electric mantle heater (Tecnovetro, Pisa, Italy). A sample (100 g) of air-dried material was hydrodistilled for 3 h. The extraction yield was 0.09 % *w/w*.

### 4.3. Gas Chromatography–Mass Spectrometry Analyses and Compounds Identification

The hydrodistilled EO of *A. membranacea* was diluted to 5% in HPLC-grade *n*-hexane and then injected into a Varian CP-3800 GC-MS apparatus (GC-EI-MS, Varian, Inc. Palo Alto, CA, USA) equipped with a DB-5 capillary column (30 m × 0.25 mm; coating thickness 0.25 μm) and a Varian Saturn 2000 ion trap mass detector (Varian, Inc. Palo Alto, CA, USA). Analytical conditions were as previously reported [40]. The identification of the constituents was based on the comparison of the retention times with those of authentic samples, comparing their linear retention indices relative to the series of *n*-hydrocarbons, commercial libraries such as NIST 14 and ADAMS and a homemade mass-spectral library of pure compounds were used to compare the mass spectra [41,42].

### 4.4. Chemicals and Reagents

1,8-Cineole was kindly gifted by Mr. Mohammed Khair Al Halabi, United Tetra Group for Medical and Scientific Supplies, Jordan. Doxorubicin and all reagents were purchased from Sigma–Aldrich, St. Louis, MO, USA.

### 4.5. Cell Culture

Three human cancerous (MCF7, A2780 and HT29) and one fibroblast (MRC5) cell lines were used in this study. Cancer cells were maintained in RPMI-1640, and MRC5 cells were maintained in EMEM media. All media were supplemented with FBS (10%), sodium pyruvate (1 mM), L-glutamine (2 mM) and penicillin/streptomycin (1%). Cell cultures were kept on 5% CO_2_ in humidified incubator at 37 °C.

### 4.6. Cytotoxicity Assay

Cells were seeded in 96-well plates and exposed to the essential oil of *A. membranacea* at concentrations up to 100 μg/mL for 72 h. 1,8-cineole and doxorubicin concentrations were made up to 50 µM also for 72 h. MTT assay was then performed as previously reported [43]. Absorbances were measured using a multi-plate reader at a wavelength 550 nm. IC_50_ values were determined by calculated the oil concentration-induced 50% reduction in the absorbance compared to untreated control.

### 4.7. Induction of Apoptosis Assay

The induction of apoptosis activity of *A. membranacea* EO and 1,8-cineole was investigated by annexin V FITC/Propidium iodide protocol as described in the literature [44]. Briefly, A2780 cells were seeded at 1 × 10^5^ cells/well in 6-well plate overnight before treatment with either *A. membranacea* EO or 1,8-cineole at four concentrations, ranging from 0 to 50 μg/mL, or 0–1 µM, respectively (representing IC_50_: 0, ×1, ×2 and ×4). Following treatment, cells were collected (including the supernatant), washed with ice-cold PBS and then incubated for 2 min in binding buffer (100 µL) and annexin V FITC (10 μL), at room temperature in the dark. Additional 400 μL of binding buffer and 10 μL PI were then added. The early apoptotic, late apoptotic, and necrotic cell populations were analyzed by flow cytometry (FC500, Beckman Coulter, Brea, CA, USA), based on 20,000 events per sample.

### 4.8. Perturbation of the Cell Cycle

A2780 cells were seeded at 1 × 10^5^ cells/well in 6-wellplate overnight before being treated with either *A. membranacea* or 1,8-cineole essential oils at four concentrations ranged from 0 to 50 μg/mL, or 0 to 1 µM, respectively. Cells were then suspended by trypsin-EDTA and spun at 200× *g* for 5 min. Collected cell pellets were washed in ice-cold PBS before they were fixed in 70% ice-cold ethanol overnight. The cell cycle analysis was then performed and analyzed for 20,000 events per sample, using flow cytometry (FC500, BC, USA), following the protocol described elsewhere [45,46].

### 4.9. Statistical Analysis

Statistical differences of samples, compared to untreated control cells, were assessed by a one-way ANOVA with the Tukey’s post-hoc multiple comparison test (GraphPad Prism Version 5, GraphPad Software, San Diego, CA, USA). *p* < 0.05 (*), *p* < 0.01 (**) and *p* < 0.001 (***) were taken as significant.

## Figures and Tables

**Table 1:** Complete composition of *A. membranacea* essential oil (EO).

Constituents	l.r.i.^a^	Relative Abundance (%)
(*E*)-2-hexenal	855	1.7
1-hexanol	868	0.4
heptanal	900	0.4
(*E*,*E*)-2,4-hexadienal	910	tr ^b^
α-thujene	931	tr
α-pinene	939	0.7
camphene	953	tr
(*Z*)-2-heptenal	959	0.2
benzaldehyde	961	0.8
1-heptanol	969	tr
sabinene	976	tr
1-octen-3-ol	978	0.2
6-methyl-5-hepten-2-one	985	0.7
2,3-dehydro-1,8-cineole	991	1.3
octanal	1002	1.6
α-phellandrene	1005	0.4
(*E*,*E*)-2,4-heptadienal	1015	0.4
α-terpinene	1018	0.2
*p*-cymene	1027	0.3
1,8-cineole	1035	21.7
(*Z*)-β-ocimene	1041	tr
3-octen-2-one	1042	tr
phenyl acetaldehyde	1045	1.5
(*E*,*E*)-3,5-octadien-2-one	1090	0.4
linalool	1099	1.1
α-thujone	1102	2.3
*cis*-*p*-menth-2-en-1-ol	1123	2
α-campholenal	1127	tr
*cis*-*p*-mentha-2,8-dien-1-ol	1139	tr
*trans*-pinocarveol	1140	3.1
camphor	1145	1.6
(*E*)-2-nonenal	1162	0.7
pinocarvone	1164	1.0
borneol	1166	4.3
4-terpineol	1178	0.9
*p*-cymen-8-ol	1185	0.3
cryptone	1186	tr
α-terpineol	1190	1.4
(*Z*)-4-decenal	1194	2.2
myrtenal	1195	0.8
safranal	1200	0.4
decanal	1205	2.5
*trans*-carveol	1219	tr
β-cyclocitral	1223	0.3
isobornyl formate	1233	0.3
piperitone	1254	2.1
linalyl acetate	1258	0.7
*cis*-chrysanthenyl acetate	1263	tr
geranial	1272	tr
isobornyl acetate	1286	0.3
thymol	1291	0.3
carvacrol	1300	0.4
(*E*,*E*)-2,4-decadienal	1316	0.2
methyl decanoate	1326	tr
hexyl tiglate	1333	0.7
*trans*-piperitol acetate	1346	tr
α-terpinyl acetate	1351	tr
eugenol	1358	tr
neryl acetate	1367	tr
α-copaene	1376	tr
(*E*)-β-damascenone	1382	0.9
*n*-tetradecane	1400	0.2
methyl eugenol	1403	tr
dodecanal	1408	tr
β-caryophyllene	1418	0.4
(*E*)-α-ionone	1428	0.3
cabreuva oxide A	1447	tr
(*E*)-geranyl acetone	1454	1.7
β-santalene	1462	1.2
cabreuva oxide D	1480	0.8
germacrene D	1485	0.9
(*E*)-β-ionone	1485	0.6
*cis*-β-guaiene	1492	0.9
bicyclogermacrene	1494	0.4
*n*-pentadecane	1500	tr
tridecanal	1518	0.3
myristicin	1520	3.3
7-*epi*-a-selinene	1522	0.3
α-cadinene	1538	2.6
ledol	1565	0.3
*trans*-nerolidol	1566	0.5
spathulenol	1576	2.7
caryophyllene oxide	1581	0.8
*n*-hexadecane	1600	0.4
β-oplopenone	1606	0.4
dill apiole	1621	0.6
τ-cadinol	1641	0.4
β-eudesmol	1649	0.7
intermedeol	1667	0.3
*n*-heptadecane	1700	tr
pentadecanal	1717	0.2
hexahydrofarnesylacetone	1845	tr
(3*Z*)-cembrene A	1959	0.4
linoleic acid ethyl ester	2160	2.3
1-pentacosene	2400	3.8
*n*-pentacosane	2500	0.6
Monoterpene hydrocarbons		1.6
Oxygenated monoterpenes		45.9
Sesquiterpene hydrocarbons		6.7
Oxygenated sesquiterpenes		6.9
Diterpene hydrocarbons		0.4
Apocarotenes		4.2
Phenylpropanoids		3.9
Other non-terpene derivatives		22.4
Total identified (%)		92.0

^a^ Linear retention indices on a DB-5 capillary column; ^b^ Traces, < 0.1%.

**Table 2:** Cytotoxic activity of *A. membranacea* essential oil against three cancer cell lines and one normal fibroblast (MTT 72 h, IC_50_ ± SD μg/mL).

MCF7	A2780	HT29	MRC5
50.86 ± 10.14	12.99 ± 2.96	14.02 ± 4.89	49.25 ± 1.27

**Table 3:** Cytotoxic activity of 1,8-cineole and doxorubicin against A2780 and MRC5 cells (MTT 72 h, IC_50_ ± SD μM)**.**

	A2780	MRC5
1,8-Cineole	0.26 ± 0.04	10.50 ± 1.70
doxorubicin	0.14 ± 0.02	0.21 ± 0.03
